# New generation continuous cardiac output monitoring from carbon dioxide elimination

**DOI:** 10.1186/s12871-019-0699-5

**Published:** 2019-02-26

**Authors:** Philip J. Peyton, Mats Wallin, Magnus Hallbäck

**Affiliations:** 1 Anaesthesia, Perioperative and Pain Medicine Unit, Melbourne Medical School, University of Melbourne; Department of Anaesthesia, Austin Health, Heidelberg, Vic 3084 Australia; 2 Maquet Critical Care, AB, Rontgenvagen 2, S-17154 Solna, Sweden; 3 Karolinska Institute Department of Physiology and Pharmacology, Section of Anesthesiology and Intensive Care, Stockholm, Sweden

**Keywords:** Monitoring, Carbon dioxide, Cardiac output, Perioperative

## Abstract

**Background:**

There is continuing interest among clinicians in the potential for advanced hemodynamic monitoring and “goal directed” intravenous fluid administration guided by minimally-invasive cardiac output measurement to reduce complication rates in high risk patients undergoing major surgery. However, the adoption of the available technologies has been limited, due to cost, complexity and reliability of measurements provided. We review progress in the development of new generation methods for continuous non-invasive monitoring of cardiac output from measurement of carbon dioxide elimination in ventilated patients using the Differential Fick method.

**Main text:**

The history and underlying theoretical basis are described, and its recent further development and implementation using modern generation anesthesia monitoring and delivery systems by two separate but parallel methods, termed “Capnotracking” and “Capnodynamics”. Both methods generate breath-by-breath hands-free cardiac output monitoring from changes in carbon dioxide elimination produced by automatic computerized modulation of respiratory rate delivered by an electronic ventilator. Extensive preclinical validation in animal models of hemodynamic instability, with implanted ultrasonic flow probes for gold standard reference measurements, shows this approach delivers reliable, continuous cardiac output measurement in real time. The accuracy and precision of measurement by the Capnodynamic method were maintained under a wide range of both hemodynamic and respiratory conditions, including inotropic stimulation, vasodilatation, hemorrhage, caval compression, alveolar lavage, changes in tidal volume and positive end-expiratory pressure, and hypercapnia, with only brief derangement observed in a model of lower body ischemia involving release of prolonged aortic occlusion by an intra-aortic balloon. Phase 2 testing of a Capnotracking system in patients undergoing cardiac surgery and liver transplantation has achieved a percentage error of agreement with thermodilution of +/− 38.7% across a wide range of hemodynamic states.

**Conclusions:**

Progress in development of these technologies suggest that a robust, automated and reliable method of non-invasive cardiac output monitoring from capnography is close at hand for use in major surgery and critical care. The great advantage of this approach is that it can be fully integrated into the anesthesia machine and ventilator, using components that are already standard in modern anesthesia and intensive care workstations, and should be virtually hands-free and automatic.

## Background

### The need for better cardiac output monitoring options

Over the last 25 years, progress in technologies for perioperative measurement of cardiac output (CO) has been largely driven by the potential for advanced hemodynamic monitoring to deliver improvements in patient outcomes in major surgery. Much of this interest among clinicians and researchers relates to “goal directed” intravenous fluid administration, informed by our understanding of the relationship between circulatory volume status and stroke volume based on the Starling curve [1–5]. This is manifested clinically as “fluid responsiveness”, where cardiac output can be increased by greater ventricular preload to a maximal point representing “optimal” volume status. The potential clinical implications of this were articulated by Bellamy, who drew a theoretical U-shaped curve relating optimal fluid resuscitation and tissue perfusion to minimizing complication rates in surgical patients [6]. Recent large trial data have supported, but not yet confirmed, this in high risk patients undergoing major abdominal surgery [7]. In addition to fluid and volume titration, cardiac output measurement provides other important benefits such as improved diagnostic capability to help manage hypotension in the anesthetized patient and assist appropriate combination of fluid and vasopressor administration by calculation of systemic vascular resistance (SVR) from measurement of both systemic perfusion pressure (the difference between mean arterial pressure MAP and central venous pressure CVP) and blood flow (Eq. 1).

1 $$ \left( MAP- CVP\right)= CO\ X\  SVR $$

Invasive cardiac output measurement from right heart thermodilution using the pulmonary artery catheter (PAC) became widespread practice in cardiac surgery forty years ago and remains routine in many centers worldwide. However the overall risk-benefit of the PAC has been difficult to demonstrate, and its invasiveness prevented its adoption in routine practice outside of the most challenging and high risk surgical populations. Nevertheless, the desire to generalize the potential benefits of advanced hemodynamic monitoring to a wider population has driven an explosive growth in the range of new less invasive alternatives. Several different physiological and bioengineering principles have been exploited to achieve this, including pulse pressure waveform interpretation, Doppler measurement of blood flow velocities in the great vessels, and thoracic electrical bioimpedance and velocimetry and related technologies. Testing and validation of these devices in the clinical environment has generally used thermodilution as the reference standard, and has usually been conducted in the setting of elective cardiac surgery [8]. Interpretation and generalization of the findings of these validation studies is often made difficult by the narrow test population, as well as the imperfect precision of cardiac output measurement by thermodilution under real world clinical conditions [9, 10].

Despite this growth in available technologies, however, the penetration of minimally-invasive cardiac output measurement into routine patient monitoring in major surgery remains relatively poor, and largely the province of enthusiasts and researchers [11]. There are a numbers of reasons for this. The available technologies, embodied as either stand alone devices or modules incorporated into anesthesia platforms, add clutter and complexity to the anesthesiologist’s work space. Peripheral disposable components can add significant cost to anesthetic care. Reservations remain about the reliability of measurements provided, particularly when employed outside of the “comfort zone” of elective surgery where they have been tested, such as in unstable or critically ill patients where the information they provide should be of greatest clinical benefit. Published data from the most widely studied generic technologies show agreement with thermodilution (percentage error) under clinical conditions of +/− 40–45% [8]. In the absence of firm data from large randomized trials that these monitors improve patient outcomes, these drawbacks discourage the clinician from their use and remain a barrier to the widespread adoption of advanced hemodynamic monitoring and fluid optimization [11].

### History of cardiac output measurement from carbon dioxide elimination

One of the oldest non-invasive techniques for measurement of cardiac output is the Differential Fick method using measurement of carbon dioxide (CO_2_) elimination. The classical principle relating lung gas exchange to pulmonary blood flow (PBF) was described for oxygen by Adolph Fick in 1870 and became a cornerstone of physiology [12, 13]. The mass balance principle is equally valid for other respired gases such as CO_2_.

2 $$ PBF=\frac{\dot{V}{co}_2}{\left({Caco}_2-C\overline{v}{co}_2\right)} $$

where $\dot{V}{co}_2$ is the measured rate of CO_2_ elimination and *Caco*_2_ and $C\overline{v}{co}_2$ are the content of CO_2_ in arterial and mixed venous blood respectively. *Caco*_2_ can be estimated non-invasively by measurement of alveolar or end-tidal CO_2_ partial pressure *P*_*E*_'_*CO*2_ with knowledge of the solubility coefficient *s* of CO_2_ in blood. However, practical application of the Fick principle was limited by the need to measure mixed venous blood content ($C\overline{v}{co}_2$). Methods such as rebreathing and breath holding maneuvers have been explored over the years but not entered routine clinical practice [14].

The Differential Fick method, first described by Gedeon et al in Sweden in 1980, cleverly overcame this problem in ventilated subjects by using a step change in lung alveolar ventilation to change measured $\dot{V}{CO}_2$ and *P*_*E*_'_*CO*2_ while $C\overline{v}{co}_2$ remained constant [15].

3 $$ PBF=\frac{\dot{V}{CO}_2i-\dot{V}{CO}_2j}{s.\left({PE}^{\hbox{'}}{CO}_2i-{PE}^{\hbox{'}}{CO}_2j\right)} $$

where *i* and *j* are measurements made at equilibrium before and after the step change in ventilation. Importantly, the measurements needed to be completed within a window of 45 s or so, to avoid error from alteration in $C\overline{v}{co}_2$ that would follow the transient change in pulmonary CO_2_ elimination induced by the maneuver.

In Gedeon’s original animal study, the ventilation change was generated by different respiratory rates delivered by a modified ventilator in a way which held the duration of expiration constant. The authors felt this pattern would have less effect on lung mechanics, particularly on alveolar deadspace which affects the relationship between alveolar (end-tidal) and arterial CO_2_ partial pressures, and thereby could confound the calculation of PBF.

Instead, the method was pursued and successfully exploited clinically by development of the partial CO_2_ rebreathing technique, where the change in alveolar ventilation was made using a partial rebreathing loop with an automated valve, placed near the mouthpiece of the breathing circuit, which opened to produce a sudden increase serial deadspace in the breathing system, and drop in effective alveolar ventilation. This approach allowed for the method to be implemented in a stand alone device, the NICO (Novametrix, USA), which was made commercially available in the 1990s, needing no manipulation of ventilator settings to achieve a measurement of cardiac output. Given the relatively limited sophistication of most anesthesia machines 25 years ago, with little ability to integrate communication between the patient monitor and ventilator, this was certainly an appropriate course to take. Later iterations of the device (NICO_2_, Respironics, USA, and NM3, Philips Corporation, USA) have followed using the same basic design. While its major limitation was that it is only able to be used in ventilated patients, the method delivered intermittent PBF measurements every 3–4 min, with a period of restabilization of $C\overline{v}{co}_2$ in between each rebreathing maneuver [16]. The theoretical sources of error with the method have been systematically explored [17], but the accuracy and precision of the partial CO_2_ rebreathing method was comparable to other minimally invasive technologies that have followed [8]. However, the clinical adoption of the partial CO_2_ rebreathing technique has been limited and it increasingly has become overlooked as an alternative for advanced hemodynamic patient monitoring.

## Modern implementation of the differential CO_2_ Fick method

Advances in design of modern anesthesia monitoring and delivery platforms now offer a real opportunity to revisit the implementation of the Differential Fick method using CO_2_ elimination. Fully integrated software controlled patient ventilators are now a routine component of modern anesthesia machines, and the original vision of Gedeon to simply modulate ventilatory pattern to generate the required inputs to calculate PBF is no longer a technological barrier. The immediate challenge has only been to define the optimal method to do so. The promised result is delivery of automatic, non-invasive, continuous cardiac output monitoring in any ventilated patient, which is essentially hands-free, with little or no requirement for added expense or complexity to the normal conduct of anesthesia. This aim has been pursued independently by two research groups on opposite sides of the globe over recent years.

### Capnotracking

Peyton et al published a 2006 study in a sheep model of hemodynamic instability, examining the relationship between cardiac output changes and fluctuations in CO_2_ elimination induced by continuous six breath alternating changes in tidal volume generated by a software controlled ventilator. Importantly, cardiac output was measured beat-by-beat in real time using an invasive gold standard in the form of an indwelling transit time ultrasonic flow probe (Transonics, USA) [18]. Peyton in 2008 proceeded to a clinical study at the Austin Hospital in Melbourne, Australia, in 24 patients undergoing cardiac surgery using a modification of the original method of Gedeon et al and Eq. 3*.* An automated change in the respiratory rate delivered by a modified ventilator (Ohmeda 7800, Datex-Ohmeda, Finland) under computer control was used, but by varying only the duration of the end-expiratory pause instead, to further minimize physiological sources of error [19]. This was followed by a larger Phase 2 single centre clinical study published in 2012 of a method termed “Capnotracking” which provided continuous cardiac output measurement [20]. The method consisted of a “calibration maneuver” involving automated periodic changes in ventilator rate similar to those tested in their 2008 study, using Eq. 3 to obtain a baseline measurement of PBF, and repeated every 30 min. In the intervening periods of constant rate and tidal volume delivery, breath-by-breath cardiac output monitoring was obtained using a “continuity equation” (Eq. 4) that related changes in measured CO_2_ elimination with each breath to changes in PBF, and which was consistent with theoretical and animal studies previously published by Breen and Isserles [18, 21, 22].

4 $$ {PBF}_k= PBFi.{\left(\frac{\dot{V}{co}_2k}{\dot{V}{co}_2i}\right)}^2 $$

where *i* and *k* are measurements made at baseline and any current breath *k* respectively. The method also estimated effective lung volume (ELV) for CO_2_ at baseline to correct for alveolar washin and washout with each change in respiratory rate, and included adjustments for physiological sources of error including non-invasive shunt estimation and correction for patient temperature and hemoglobin concentration that affect the solubility of CO_2_ in blood [23]. The method was successfully tested in 77 patients undergoing either cardiac surgery or liver transplantation, which encompass both hypo- and hyperdynamic circulatory states, providing a demanding test of the linearity and reliability of the method under a wide range of cardiac output values. Percentage error of the method relative to bolus thermodilution was +/− 44.2%, which was similar to other more cumbersome minimally invasive techniques [8].

### Capnodynamics

**Fig. 1:**
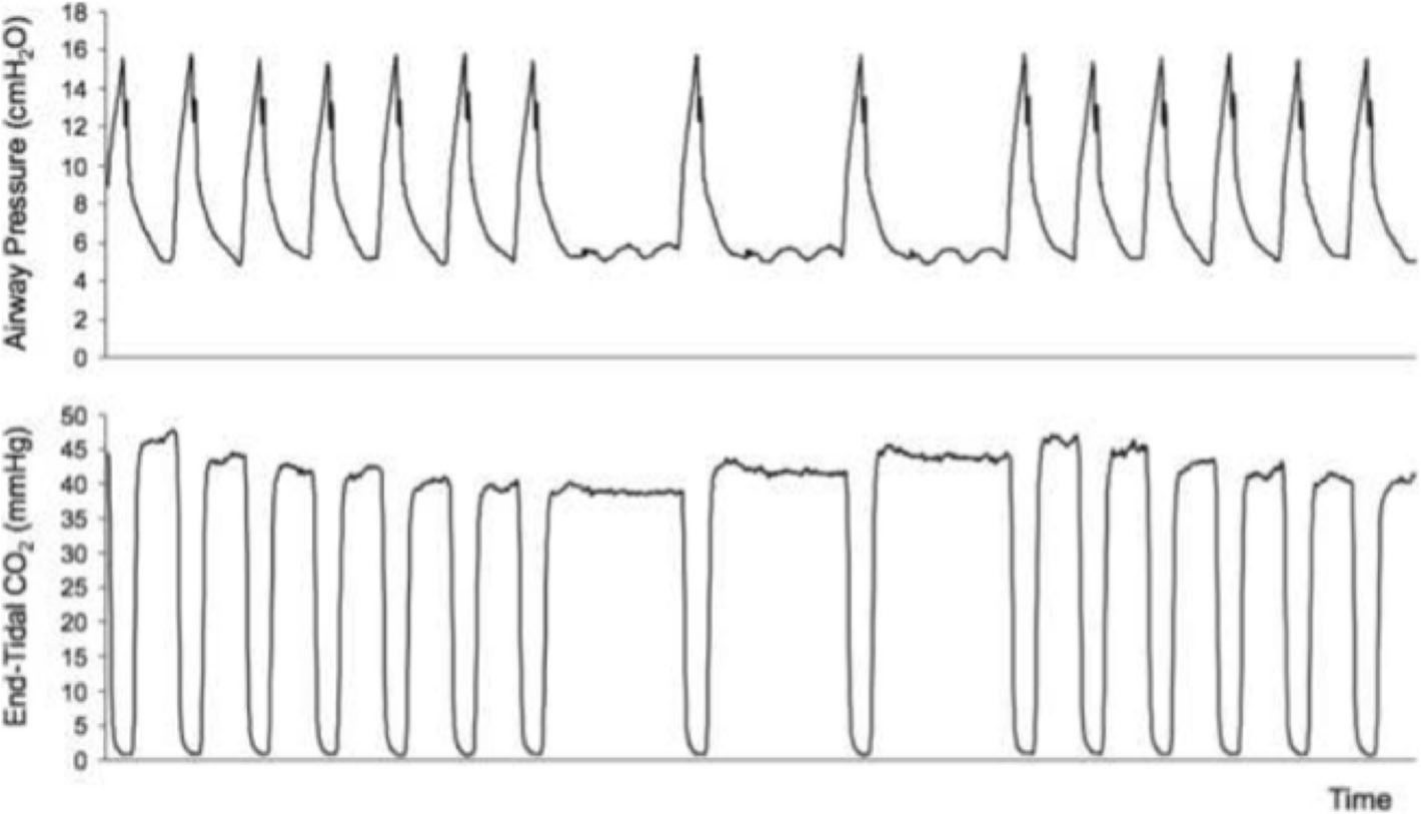
Examples of the pattern of change in respiratory rate implemented in the Capnodynamic method, and the resulting cyclic changes in end-tidal CO_2_ concentration that are the inputs for Eq. 5. A similar pattern is employed in the calibration phase of the Capnotracking method using Eq. 3

In Sweden a group sponsored by Maquet Critical Care AB in collaboration with the Karolinska Hospital, Stockholm, developed a system based on fluctuations in alveolar ventilation by automated change in rate and inspired-expired ratio similar to that originally tested by Gedeon et al, but on a continuous cyclic basis, which they termed “Capnodynamics” (Fig. 1). This cyclic breathing pattern provides multiple inputs for each variable in the Differential Fick equation, to obtain a more robust, overdetermined solution for effective pulmonary blood flow (EPBF) with each new breath [24]. This is shown in Eq. 5. The mathematics of the method will continually correct for sources of error arising from changes in the difference between measured end-tidal and arterial CO_2_ partial pressures that accompany changes in pulmonary alveolar deadspace, such as occur with changes in delivered tidal volume, or pathology such as pulmonary embolism. This is a theoretical source of error with Capnotracking where changes in alveolar deadspace will tend to magnify the response to real changes in pulmonary perfusion (Eq. 4). Eq. 5 also calculates ELV for CO_2_ continually, which may have additional benefits for respiratory management in critical care [25].

5 $$ ELV\cdot \left({{F_{ACO}}_2}^n-{{F_{ACO}}_2}^{n-1}\right)= EPBF\cdot \Delta {t}^n\cdot \left(C\overline{v}{co}_2- Cc{{{\hbox{'}}_{CO}}_2}^n\right)-{{V_{CO}}_2}^n $$

where *ELV* is effective lung volume containing CO_2_ at the end of expiration, *EPBF* is effective pulmonary blood flow, *FACO*_*2*_ is alveolar CO_2_ fractional concentration, *Cc’CO*_2_ is lung capillary CO_2_ content (calculated from *FACO*_*2*_ and the CO_2_ dissociation curve), *VCO*_*2*_^*n*^ is the volume of CO2 eliminated by current breath *n* and *Δt*^*n*^ is the current breath cycle time (min).

**Fig. 2:**
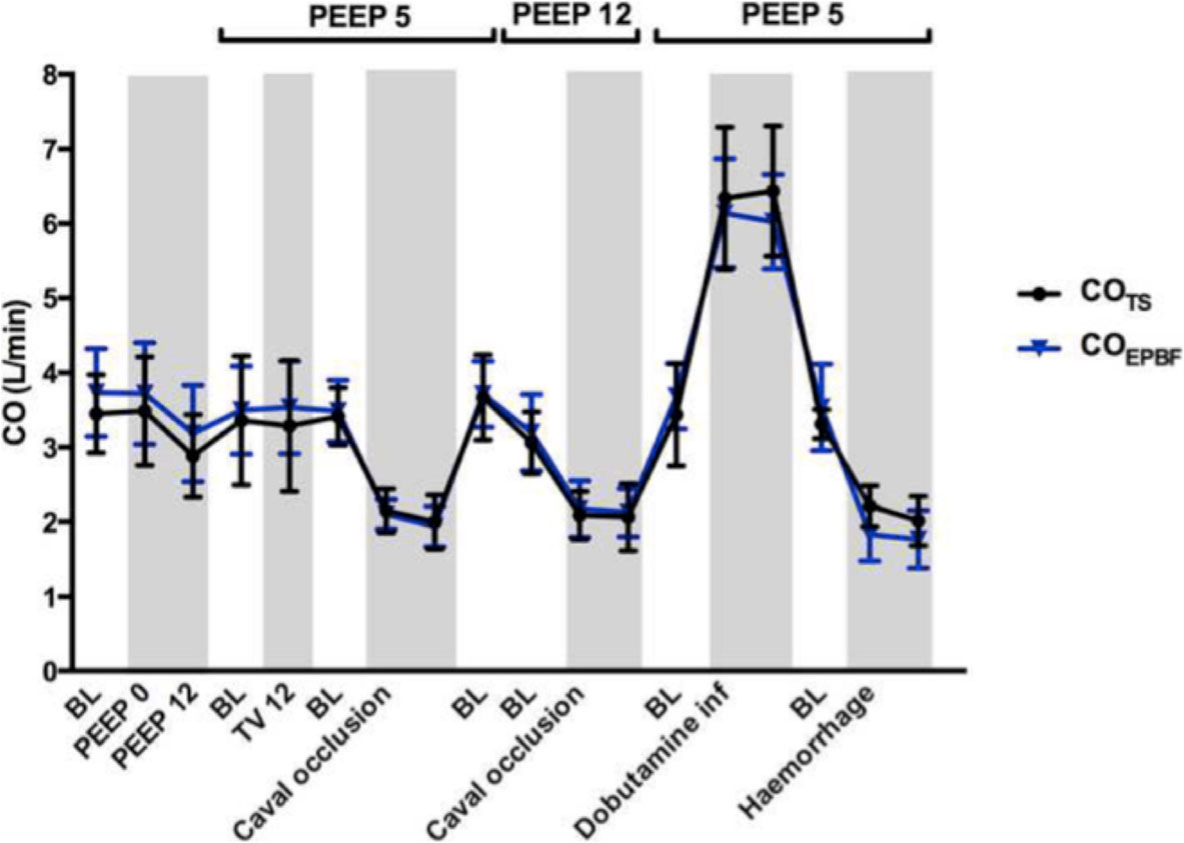
Data from a pig model using an ultrasonic transit time flow probe as reference standard (CO_TS_) showing the performance of a prototype Capnodynamic system for continuous measurement of cardiac output (CO_EPBF_) during a range of circulatory and ventilatory interventions [28]. The interventions included PEEP (positive end-expiratory pressure) changes, tidal volume increased to 12 mL/kg (TV 12), caval occlusion, dobutamine infusion and induced hemhorrage. BL = baseline

**Fig. 3:**
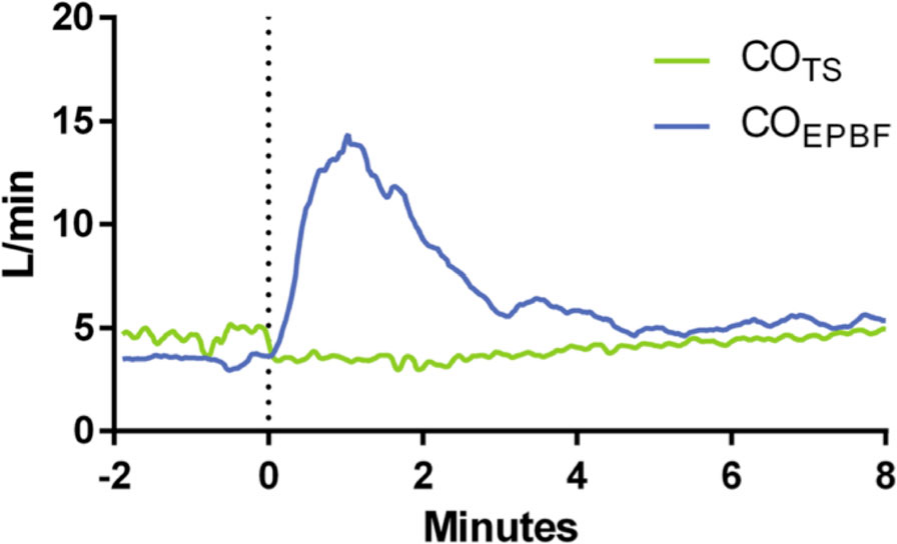
Transient error in Capnodynamic measurement of cardiac output (CO_EPBF_) compared to ultrasonic transit time flow probe (CO_TS_) after release of an aortic occlusive balloon in an animal model of prolonged lower body ischemia

**Fig. 4:**
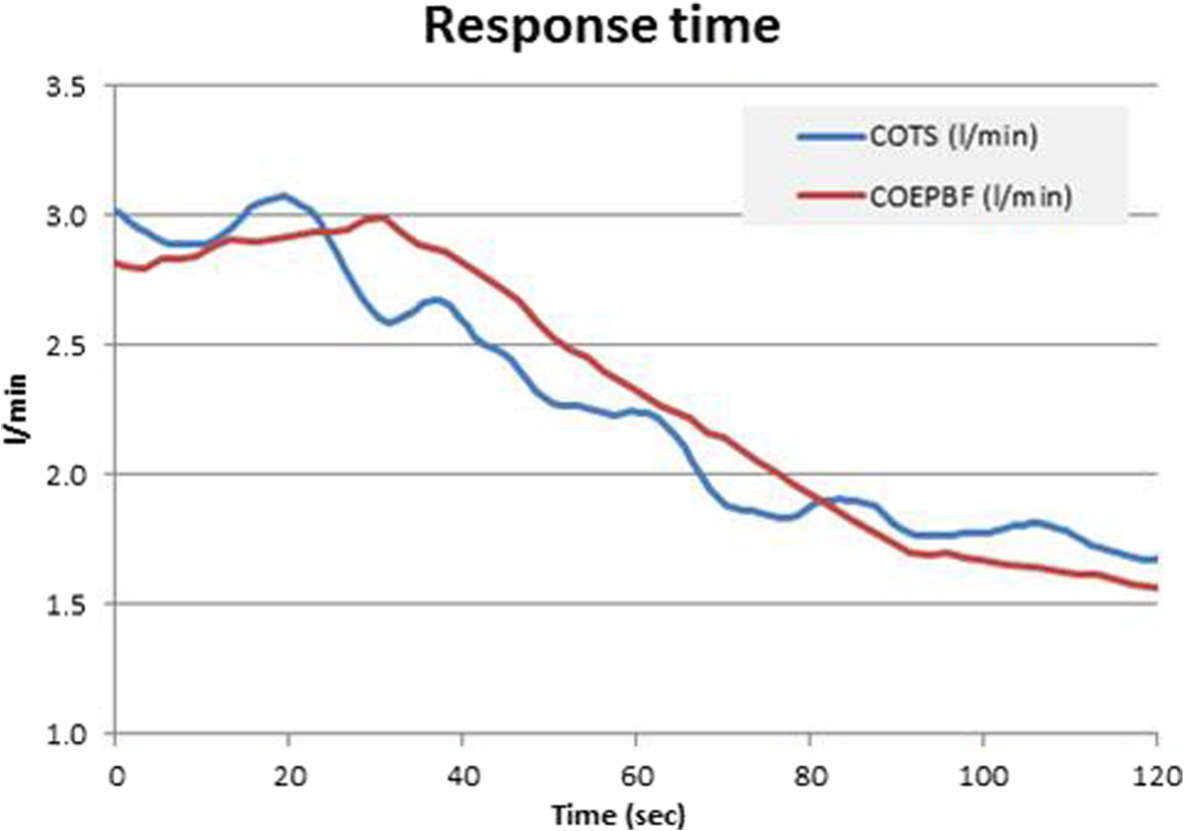
Typical response time of Effective pulmonary blood flow measured by the Capnodynamic method (CO_EPBF_) to a sudden induced fall in cardiac output measured by the ultrasonic transit time flow probe (CO_TS_) in a study in pigs [24]

**Fig. 5:**
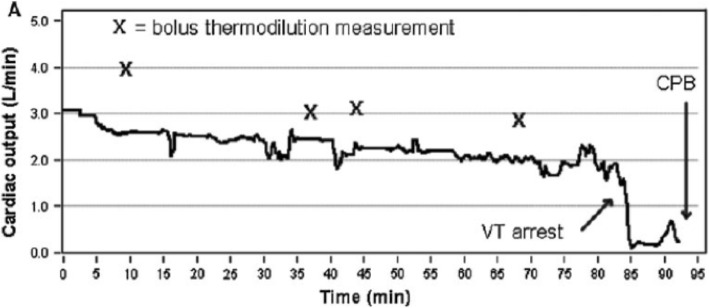
An example of the response of the Capnotracking method to a sudden collapse in cardiac output following an unexpected episode of ventricular tachycardia (VT arrest) during internal mammary artery harvesting in a patient undergoing elective coronary artery bypass grafting, with subsequent urgent run onto cardiopulmonary bypass (CPB) [20]

Using a prototype involving a modified electronic ventilator (Servo-i, Maquet, Sweden), and also using an indwelling transit time ultrasonic flow probe for a gold standard reference cardiac output measurement, they undertook an extensive program of preclinical testing of the method in a pig model. This allowed them to test it under a wide range of both hemodynamic and respiratory conditions, including inotropic stimulation, vasodilatation, hemorrhage, caval compression, alveolar lavage, changes in tidal volume and positive end-expiratory pressure (PEEP), and hypercapnia, which might potentially confound the reliability of measurement [24–27]. Interestingly, accuracy and precision obtained were found to be best where cyclic change in end-expiratory pause, similar to that validated by Peyton et al, were used, as shown in Fig. 1 [28]. They demonstrated impressive preservation of accuracy and precision of measurement relative to the flow probe across these conditions (Fig. 2), with only brief derangement observed in a model of lower body ischemia involving release of prolonged aortic occlusion by an intra-aortic balloon placed just underneath the diaphragm (Fig. 3) [27]. The response time of the method to sudden changes in cardiac output in their pig model is illustrated in Fig. 4. This underpins the clinical response observed by Peyton et al using Capnotracking in cardiac surgery patients, where its potential value as a real time monitor in a sudden unexpected crisis is shown in Fig. 5.

The fidelity of the Capnodynamic method in strenuous preclinical testing raises the question of why changes in $C\overline{v}{co}_2$ induced by the changes in ventilatory rate and CO_2_ elimination do not invalidate the measurement, in the way first expected by Gedeon and assumed in subsequent embodiments of the Differential CO_2_ Fick method such as the NICO_2_. The answer lies in the real response of $C\overline{v}{co}_2$ in the presence of continuous cyclic fluctuations in alveolar ventilation. Clearly, where ventilation is increased or decreased for a sustained period of time, CO_2_ elimination will increase or decrease in response until a new steady state is achieved with an alveolar and mixed venous CO_2_ partial pressure that is lower or higher respectively. However, where the periodicity of the change is optimal, the cyclic fluctuations generated in alveolar CO_2_ partial pressure are not mirrored in real time by change in $C\overline{v}{co}_2$, due to the widely different circulatory time constants of the various organs beds that contribute to mixed venous blood flow. The fluctuations in venous CO_2_ partial pressure within each body compartment are largely evened out as they combine to form mixed venous blood. Periodicity of ventilatory changes in the order of half a minute or so would seem to achieve this satisfactorily.

### Current clinical testing

**Fig. 6:**
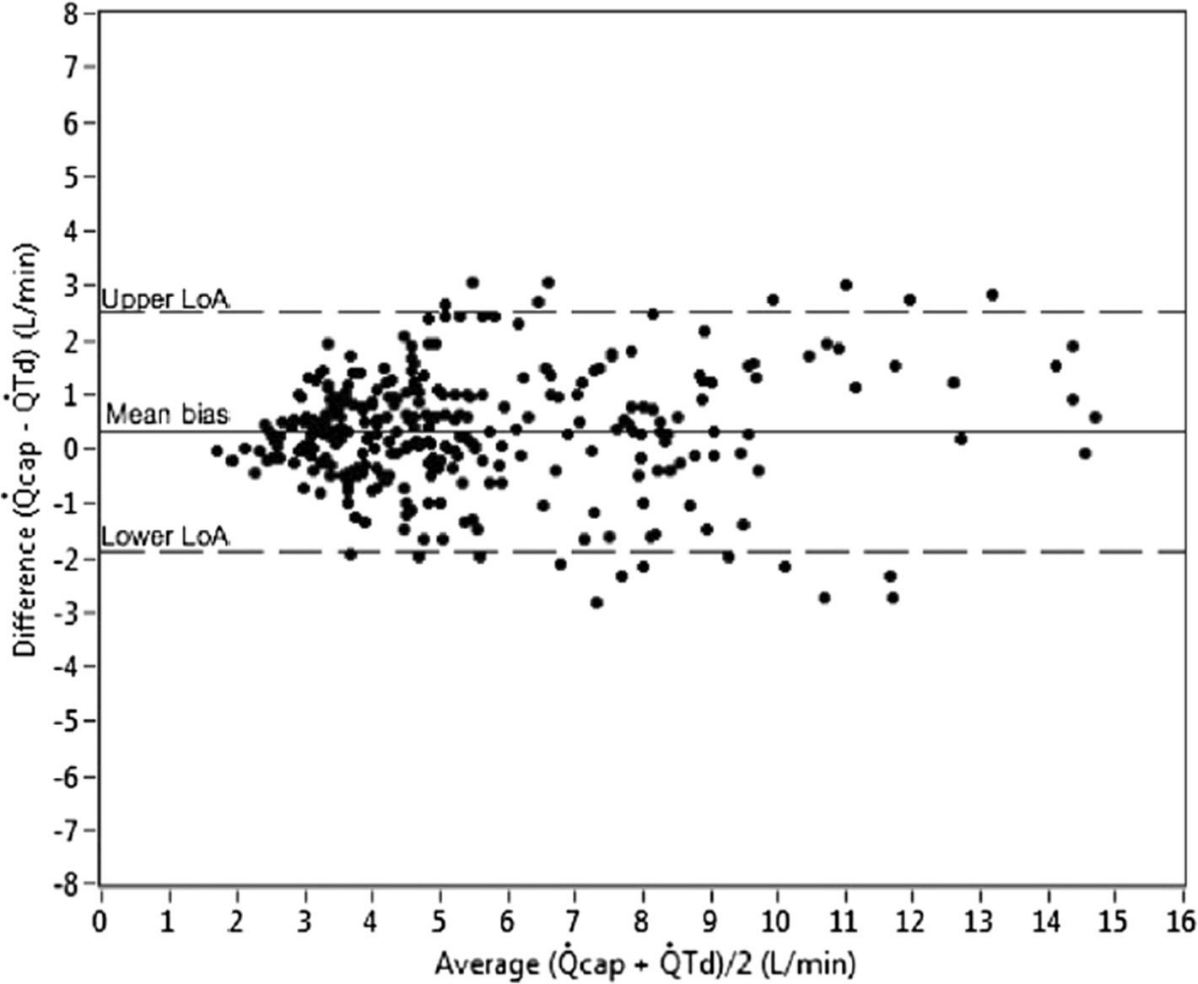
Bland Altman plot showing agreement of Capnotracking cardiac ouput measurements (*Qcap*) with thermodilution (*QTd*) in 50 patients undergoing either cardiac surgery (pre- and post cardiopulmonary bypass) or orthotopic liver transplantation. Mean bias 0.32 L/min, percentage error +/− 38.7%

**Fig. 7:**
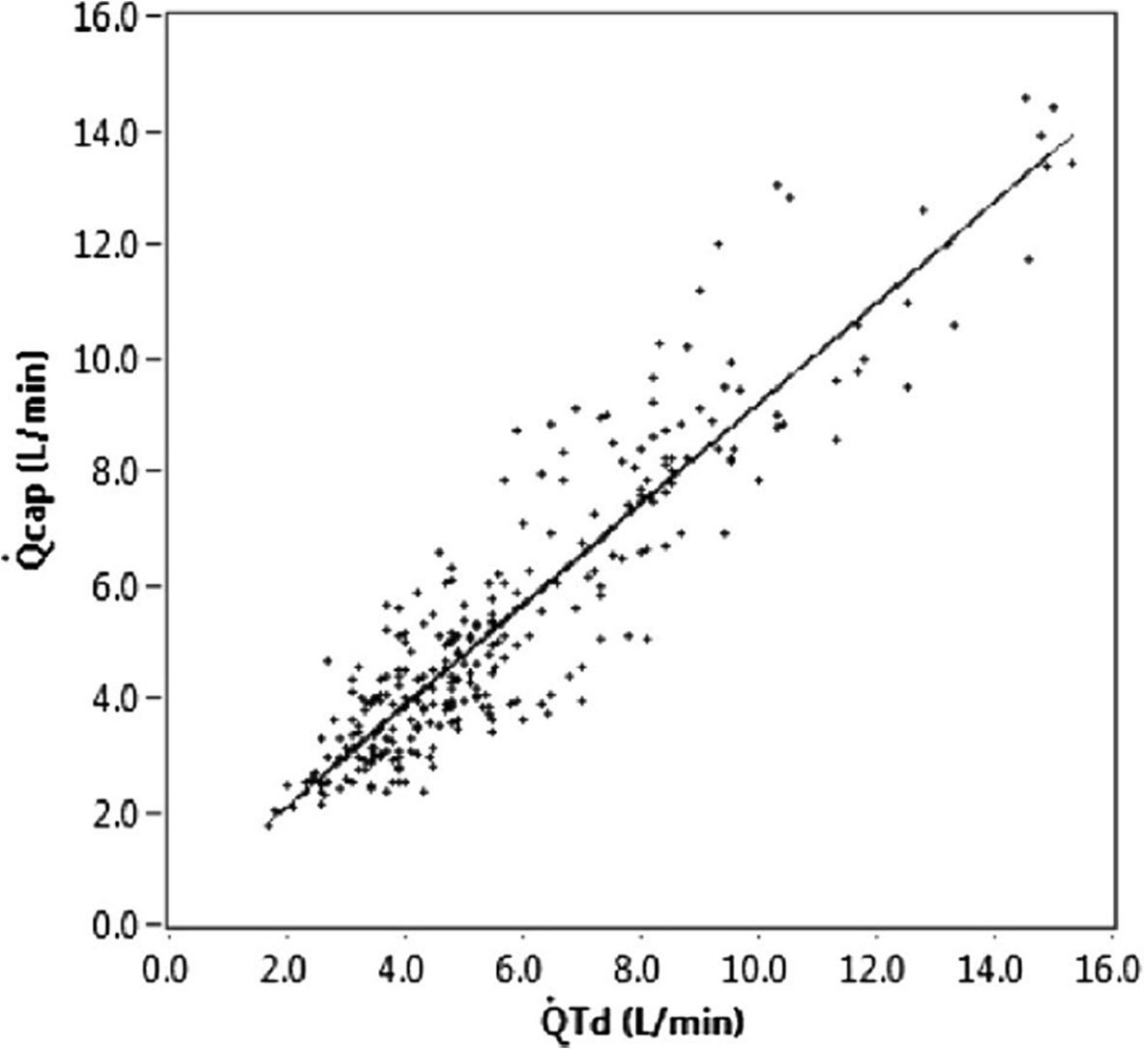
Capnotracking (*Qcap*) versus thermodilution (*QTd*), correlation plot. Intraclass correlation = 0.91

This solution to the problem of $C\overline{v}{co}_2$ change is supported by the most recent Phase 2 Capnotracking study of 50 patients by Peyton and Kozub in a similar cardiac surgery/liver transplantation population to their earlier study. Using a more prolonged “calibration” maneuver than previously employed, where a cyclic change in ventilation was pursued over at least a 2 minute period and repeated more frequently, the percentage error of the Capnotracking method relative to thermodilution was significantly reduced to +/− 38.7% across a wide range of hemodynamic states [29]. Data showing agreement with thermodilution are shown in Figs. 6 and 7.

Clinical testing of the Capnodynamic method by Maquet/Getinge is currently underway using a prototype delivery system. The first clinical study, investigating its reliability in 15 children (7.8–10.5 kg) undergoing cleft lip/palate surgery was recently published, using trans-thoracic suprasternal Doppler performed by an experienced pediatric cardiologist as the reference method [30]. Baseline agreement between Capnodynamics and Doppler was a mean bias of – 2.4% and percentage error of +/− 40%. The patients were then exposed to a PEEP step from 3 to 10 cm H_2_O (anticipated to decrease CO), and a dose of atropine (expected to increase CO by a rise in heart rate) prior to commencement of surgery. Doppler measurements confirmed the change in CO from atropine, but did not detect the anticipated change in CO from PEEP, both of which were captured by the Capnodynamics system (bias − 4.4% percentage error +/− 50%; and bias − 15.6% percentage error +/− 54% respectively).

This prompted the authors to repeat the study in piglets with a transit time ultrasonic flow probe mounted directly on the pulmonary artery, which confirmed the expected changes in CO from both PEEP and atropine with excellent agreement with Capnodynamics (percentage error +/− 31%), suggesting an inability of the Doppler method to detect the decrease in CO when PEEP was raised [30]. This has provided both interesting early clinical results and pointed to the difficulties and challenges arising from the reliability of available reference standards against which to assess any new method in the clinical environment [31].

### Limitations

The obvious limitation of this approach to cardiac output measurement, using the methodologies outlined here, is that it is restricted to patients undergoing controlled ventilation. The degree to which lung disease interferes with the reliability of measurement in the clinical setting will require exploration in future Phase 3 testing. Certainly, animal data on Capnodynamics suggests that the method is robust in the face of severe acute lung injury. Anecdotal experience with Capnotracking to date suggests that the method remains valid in patients with moderate obstructive lung disease. However, in patients with severe acute or chronic airflow obstruction with gas trapping, slowing respiratory rate by prolonging the duration of the end-expired pause can paradoxically increase CO_2_ elimination with each breath rather than simply reducing it in proportion to the fall in rate. However, this is a small subset of patients encountered in major surgery, in whom another method for cardiac output monitoring would seem an appropriate choice. Some metabolic changes may affect the accuracy of the calculation of PBF, for example, changes in hemoglobin concentration which affect the solubility of CO_2_ in blood. In the setting of significant surgical blood loss, regular measurement of hemoglobin concentration is advisable, and new techniques for continuous, non-invasive hemoglobin monitoring would seem to dovetail well with this technology.

### Future directions and implications

Further testing of the Capnodynamic method across a range of circulatory states and clinical environments is ongoing. The excellent results from rigorous preclinical testing combined with the encouraging results of Capnotracking in the clinical setting suggest that a robust, reliable non-invasive method of continuous cardiac output measurement from capnography is close at hand for use in major surgery and critical care. The great advantage of this approach is that it can be fully integrated into the anesthesia machine or ICU ventilator, using components such as capnography, respirometers and electronic ventilators that are already standard in modern anesthesia and intensive care workstations, and should be virtually hands-free and automatic.

The accessibility and ease of use this offers promises to make future large trials readily achievable into the application of the method to patient care, in particular into the influence of continuous cardiac output and advanced hemodynamic monitoring on patient outcomes in major surgery. Formal clinical comparisons with other methods in reliability of assessment of fluid responsiveness for example, would be useful. However, there may be much to gain by combining the strengths of various different techniques in different situations, for example using intraoperative capnography-based measurements, alone or in combination with other methods such as pulse contour derived measurements, to obtain a more precise intraoperative measurement, which can effectively then “calibrate” postoperative measurements in the extubated patient [32]. This “hybrid” methodology may be able to deliver levels of precision and reliability in patients that consistently approach or exceed desired benchmarks in agreement with invasive standard methods such as thermodilution [33, 34].

## Conclusion

In hindsight, Gedeon’s clever idea for cardiac output measurement was too far ahead of its time, needing to wait for the infrastructure of anesthesia monitoring and delivery that was routinely available in the operating room to catch up and provide the means for its full exploitation. Furthermore, the solution to the problem of $C\overline{v}{co}_2$ change that appeared to limit the reliability of the Differential CO_2_ Fick method has in fact been “hiding in plain sight” all along. As such, we are optimistic that this is an idea whose time has finally come, and that it will be able to deliver continuous and reliable cardiac output monitoring, with an ease and accessibility previously not achievable by other techniques. This, we hope, will dramatically improve the routine penetration of advanced hemodynamic monitoring into anesthesia for major surgery and critical care, and allow future large clinical trials to explore the potential for this to deliver improvements in important patient outcomes [35].

## Abbreviations

Caco_2_: Content of CO2 in arterial blood
*Cc’CO*_2_: Lung capillary CO_2_ content
*CO*: Cardiac output
*CO*_*2*_: Carbon dioxide
Cvco_2_: Content of CO2 in mixed venous blood
*CVP*: Central venous pressure
*ELV*: Effective lung volume containing CO_2_ at the end of expiration
*EPBF*: Effective pulmonary blood flow
*FACO*_*2*_: Alveolar CO_2_ fractional concentration
*MAP*: Mean arterial pressure
*PBF*: Pulmonary blood flow
PE’CO_2_: Alveolar or end-tidal CO2 partial pressure
*PEEP*: Positive end-expiratory pressure
*SVR*: Systemic vascular resistance
Vco_2_: Rate of CO2 elimination
*VCO*_*2*_^*n*^: Volume of CO2 eliminated by current breath *n*
*Δt*^*n*^: Current breath cycle time (min)
